# Community voices: broadening participation in Science, Technology, Engineering, Mathematics, and Medicine among persons with disabilities

**DOI:** 10.1038/s41467-022-34711-w

**Published:** 2022-12-03

**Authors:** Siobhán M. Mattison, Logan Gin, Allistair A. Abraham, Megan Moodie, Feranmi Okanlami, Katherine Wander

**Affiliations:** 1 University of New Mexico, Department of Anthropology, Albuquerque, NM 87131 USA; 2 Brown University, Sheridan Center for Teaching and Learning, Providence, RI 02912 USA; 3 George Washington University, Department of Pediatrics, Washington, DC 20052 USA; 4 University of California at Santa Cruz, Department of Anthropology, Santa Cruz, CA 95064 USA; 5 University of Michigan, Family Medicine, Physical Medicine & Rehabilitation, Ann Arbor, MI 48108 USA; 6 Binghamton University (SUNY), Department of Anthropology, Binghamton, NY 13902 USA

**Keywords:** Ethics, Careers

## Abstract

Disability has too often been peripheral to efforts to widen the STEMM pipeline, hampering research quality and innovation. Inspired by change in education delivery and research collaborations during the pandemic, we offer a structure for efforts to recruit and retain disabled scientists and practitioners.

Broadening participation in STEMM (Science, Technology, Engineering, Math, and Medicine) is crucial for vitality and innovation in science, biomedicine, education, and the humanities. Disabled persons’ unique perspectives facilitate improved understanding of the social, natural, and physical world. Indeed, recent scholarship surrounding disability justice has stressed the gains and creativity that come with experiences of disability, rather than simply losses or obstacles that typically accompany definitions of disability e.g., ref. 1. Yet, persons with disabilities are both under-represented^2^ and excluded^3,4^ within STEMM. Institutional structures, both cultural and physical, impose significant barriers to participation. Employers and workplace cultures have historically designed spaces and educational processes to accommodate a segment of society whose characteristics fall within a narrow range, resulting in exclusions of individuals (including disabled) deemed non-normative^5^.

The COVID-19 pandemic overhauled the ways in which people participated in STEMM, revealing that existing structures are more malleable than previously thought. Effects on disabled scholars were both positive and negative. We focus here on experiences that highlight how disabled scholars’ needs can be met for a more inclusive research enterprise. Specifically, as “post-pandemic” transitions are underway, we argue that certain COVID-19-related adjustments should be preserved^6^. To maximize post-pandemic inclusion, we urge that provision of supports rely less on documentation of eligibility. Rather, we advocate for an approach—FAM (Flexibility, Accommodations, Modifications)—that provides broad support for all persons regardless of disability category in ways that encompass heterogeneous needs. However, in stressing some of the positive lessons learned as a result of pandemic-related adjustments, we note that this has also been a time of tremendous grief and loss, particularly for people with disabilities, who have been disproportionately vulnerable to death, alongside those from other marginalized communities^7^. Our comments are made not to erase these experiences, but to honor them through efforts to build better futures.

## Defining disability recognizes heterogeneity

Disability is a broad concept, including visible and invisible physical and psychological impairments, as well as chronic conditions and diverse life experiences that do not map neatly onto medical definitions of disability. Indeed, definitions of disability vary widely and affect not only the statistics used to characterize the status quo, but also how individuals experience and interact with disability. Disability is defined in biomedicine by “impairment”, which de-emphasizes disability’s social and environmental dimensions as well as the viewpoints of many with disabilities who do not consider themselves to be impaired^8^. Disability activists often point to the social origins of disability, by contrast, recognizing that physical and mental variability are essential features of the spectrum of human experience, but become disabling when certain portions of the spectrum are prioritized over others.

Further, disability can be described by person-first (i.e., persons with disabilities) versus identity-first (d/Disabled persons) language. The former emphasizes the individual as non-isomorphic and defined by more than one aspect of their mental/physical being whereas the latter emphasizes disability as a core feature of one’s identity. We recognize that there are many different reasons that a person might choose one or the other. We stress that neither of these is inherently more correct than another; we recommend engaging disabled colleagues, loved ones, and acquaintances to ask - and follow - their preference. Every reader is or will be impacted by disability - lack of awareness of how it affects members of one’s community is cause for reflection. This is both culturally sensitive and recognizes the vast diversity within the disabled community.

Like non-disabled persons, disabled persons are diverse, demographically and in the manifestation of disability and ability. Yet people with disabilities’ abilities are often overlooked and their talent often lost. Ableist and disableist cultures in academic and industrial settings reify ability, stigmatize disability, and continue to create insurmountable barriers to inclusion of people with disabilities^8^. For example, people with disabilities are more likely than people without disabilities to self-finance their post-graduate education, the share of disabled persons in the workforce declines as specialization levels increase^2^, and academic staff with disabilities have even less support than do disabled students and trainees^8^. People with disabilities must further manage the invisible costs of being disabled (“disability admin”^9^). They are frequently encouraged not to disclose their disabilities, limiting representation^8^. Exclusion of disabled scholars limits diversity, narrows the STEMM pipeline, and hampers scientific innovation and quality.

## Leveraging lessons learned to enable full participation in STEMM

Enabling full participation of persons with disabilities has been a priority for over thirty years, as codified by the 1990 Americans with Disabilities Act (ADA) in the US, the Disability Discrimination Act 1995 (DDA) in the UK, and prior anti-segregationist legislation (e.g., the Individuals with Disabilities Education Act (IDEA)). Accommodations provided under relevant legislation must be both “reasonable” (i.e., cost effective and “fair”) and supported by medical diagnoses and documentation. The purpose of such accommodations is to provide equal access and opportunity and to enable disabled persons to preserve the “essential functions” of positions nearly always designed for non-disabled individuals. Although well-intended, this process generates financial and emotional stress for employers and disabled persons^1^. Supervisors and disabled persons often have limited understanding of what constitutes eligibility as well as what supports are necessary and available to the broad array of individuals deemed eligible under the law. In theory, such stresses could be alleviated by shifting the emphasis from questions of eligibility and reasonableness to proactive delivery of supports, but there is little evidence that such delivery exists in most workplaces in the United States and elsewhere.

The COVID-19 pandemic demonstrated that large-scale changes in education delivery and research collaboration are possible and often beneficial for disabled persons and others facing a variety of constraints. Large swaths of the STEMM workforce successfully continued research, education, and development activities remotely or with major modifications to in-person activities, including flexible hours, modified schedules, and caps on occupancy. These changes were associated with many positive outcomes, including reduced pollution^10^, improved access via online delivery formats e.g., ref. 11, and greater work efficiency e.g., ref. 12. Many disabled people were inadvertently accommodated in ways that had previously been deemed impossible for both disabled and non-disabled individuals, including working from home, with flexible schedules, variable workloads, and delayed deadlines. While minimizing COVID-19 risk, these changes meant that people with disabilities that restrict in-person work could nonetheless attend meetings and conferences, rest and recover, and otherwise balance the demands of their conditions alongside their contributions to their employers.

As pandemic dynamics shift and demands to return to “pre-pandemic life” are underway, we anticipate losses in diversity, equity, inclusion, and accessibility for disabled researchers (and more broadly; see ref. 13). But is this necessary? What lessons from the pandemic era can inform our approach to inclusion of disabled individuals? Our strategy borrows from the field of Disability Studies to both remind readers of the moral imperative to empower all individuals to participate maximally in society^14^ and to clarify that doing so benefits disabled and non-disabled persons alike.

## FAM: a framework for improving access to and participation in STEMM

A marriage of Disability Justice and principles borrowed from Universal Design offers a set of high-level supports for people with disabilities that is cost-efficient and broadly beneficial to people with and without disabilities. A Disability Justice approach celebrates rather than laments the infinite variability in the human form, shifting the focus from individual difference to the forms of “brilliant imperfection” that disabled people bring to the world^15^. The Universal Design framework shifts responsibility for maximizing access to built and cultural environments from individuals with disabilities to wider society, with the acknowledgement that it is impossible to foresee all accommodation needs. Rather than waiting for someone who has been excluded to request accommodation, Universal Design attempts to minimize the need for requests and, when they occur, to provide flexible forms of support. Universal Design has long highlighted that changes made to accommodate disabled people (e.g., curb cut-outs for wheelchair access) often have broader benefits (e.g., for bicycle and stroller users). The marriage of these concepts suggests an important innovation to accommodation strategies—one that was frequently achieved amidst the changes COVID-19 prompted. Specifically, to support everyone’s right to participate in STEMM education^16^ and contribute to the STEMM workforce, we offer several recommendations that provide basic supports to accommodate a broad range of abilities and constraints, without the need to adjudicate disability (i.e., who “counts” as disabled^3^). The approach we advocate rests on three pillars: Flexibility; Accommodations; and Modifications (FAM).

### Flexibility

Rather than insisting all members of the workforce work in the same way, allow individuals to contribute in ways that meet their specific needs, abilities, and preferences. Hybrid workforces are clearly possible. They allow disabled and non-disabled individuals room to adjust their schedules and modes of work for their individual needs without sacrificing the overall functions of their positions. Allowing faculty to shift courses to an online or hybrid format helps scholars and students with certain disabilities to manage things like flares and treatment (for example, two of the authors work from home during medical infusions – see Supplementary Information). Providing telehealth options allows disabled clinicians to provide care and disabled patients greater access to care. Such flexibility has clear benefits for those without disabilities, as well: teaching online allows faculty with families to accommodate care demands; options to attend online can improve students’ access; telehealth minimizes travel costs and improves access to care for patients in remote areas; and providing remote or online ways to participate in academic conferences can reduce financial barriers to attendance for graduate students and contingent faculty.

Hybrid work also highlights the need for careful and on-going assessment of equitable workplace and learning formats; for example, online formats must be designed to meet neuro-diverse needs and to work optimally for those with visual and hearing disabilities. Indeed, online teaching can exacerbate certain forms of neurological and physical discomfort and impairment, causing recurrent flares. This contrast—some people with disabilities benefit from online formats, while others’ disabilities are exacerbated—highlights the need for employee agency and employer good faith: a false sense among institutions that there is widespread consensus within a disability or patient community around remote work (or any other innovation) is counterproductive to broadening participation.

Similarly, many graduate programs waived standardized testing requirements for applicants (e.g., the Graduate Readiness Exam or GRE in the US) who could not complete the exam in person. Such waivers are being retained by many programs to avoid discrimination against students whose performance and capacities are poorly reflected by such tests; such waivers may benefit people with certain learning disabilities and other people with disabilities, who face distinct stigma in STEMM due to perceptions about their intelligence^8^. The heterogeneity of disability requires forethought to maximize inclusion.

### Accommodations

Adjustments made to built and cultural environments to maximize inclusion should remain a priority post-COVID-19. This includes compliance with laws, such as accessible ramps, bathrooms, and physical spaces. Often what is most important is basic maintenance of extant resources, many of which lapse without consistent effort. Further, we emphasize that while devices are an important part of accommodating individuals with physical disabilities, we cannot stop there. Rather, accommodations must allow for inclusion of individuals with physical disabilities, as well as neurological, social, or invisible disabilities, including conditions that do not require use of assistive technologies. Examples include accommodations for hearing and visual disabilities in meetings and workplace environments, and areas for employees, faculty, and students to rest or receive unobtrusive treatments. This approach should extend further to creating workplace cultures that invite and leverage difference to meet shared objectives see ref. 17. During the COVID-19 pandemic, lecture halls were outfitted for testing and vaccination and masks were an expectation at many institutions. This illustrates that we can make rapid changes to physical spaces and social norms when we decide doing so is a priority.

### Modifications

Finally, modifications to essential duties and/or time requirements must be incorporated into our approach to diversity, equity, inclusion, and accessibility (DEIA) in order to achieve full participation of disabled people in STEMM. While it is possible for accommodations and flexibility to allow some disabled individuals to occupy the same position as non-disabled individuals, reliance on these alone is inadequate for some disabilities. People with disabilities often must devote significant time and energy just to care for themselves, including medical appointments and treatments^9^. Modifications to workplace environments and job duties allow disabled scholars to balance the demands of their disabilities against innovative and necessary contributions to research. Additionally, some people with disabilities will benefit from modified schedules or duties. One specific example on university campuses has been the ability to count summer teaching toward required course loads so that courses can be spaced more evenly throughout the year to allow for recovery periods or to avoid particularly heavy teaching periods. Structural changes—e.g., ensuring benefits eligibility for part-time workers—may be necessary to facilitate appropriate modifications. Modifications should encourage inclusion of people with disabilities at all ranks of the academic, biomedical, and scientific workforce, including in the highest positions of leadership^8^. Knowing that employees often become disabled over the course of STEMM careers, which are often lifelong, it is also essential to commit to ongoing employment and job redefinition or transfer, should the need arise.

The approach we outlined above (see summary and extensions in Table 1) offers many benefits to disabled and non-disabled individuals. At the same time, disability is frequently overlooked or deemphasized in diversity initiatives. Disability must be prioritized to avoid loss of human capital. Most institutions’ COVID-19 policies have been, and will continue to be, dynamic; the process of revisiting these policies should consider not only how COVID-19 transmission might be affected, but also how disabled people’s and others’ ability to learn and work may be affected. In alignment with federal policy (e.g., Executive Order 14035), which highlights the ongoing need to recruit and retain people with disabilities, STEMM employers should name disability as an important vector of diversity and provide funding to individuals with these identities. Grants should incentivize institutions and labs to build accessible structures and cultures. Employers’ diversity initiatives should target the recruitment and retention of diverse people with disabilities.

**Table 1:** Suggestions for systematic application of FAM principles to improve participation of disabled people in STEMM^a^

Knowledge building	• Recognize disability as central to Diversity, Equity, Inclusion, and Accessibility (DEIA) initiatives
	• Provide evidence-based training to increase awareness for rights of individuals with disabilities to promote allyship and advocacy for all faculty, staff, and students
	• Invite experts on disability to explain the landscape of disability and related scholarship
	• Distribute disability reference guides alongside orientation materials and at regular intervals thereafter
	• Avoid over-generalizations and stereotyping by incorporating the full range of disability experience in the above efforts
Systematic assessment	• Assess physical and non-physical infrastructure with reference to meeting needs of all users
	• Invite people with disabilities to lead and participate in these efforts
	• Conduct listening sessions and climate surveys to assess needs, barriers, and opportunities and act on them with reference to current needs and increased participation among currently excluded individuals
Dedicated capital	• Earmark funding to improve culture and climate surrounding disability
	• Earmark funding to improve access to STEMM research, training, and capacity-building
	• Earmark funding to understand disability
	• Earmark funding for implementation to act on assessment findings
	• Include disability-related supports in all budget projections
	• Issue contracts to entities that have high DEIA performance
	• Increase Americans with Disabilities Act and related legislation accommodations and service staff
Implementation	• Share findings of assessments widely and visibly
	• Increase research and development of high-quality, evidence-based accommodations for disabled scholars
	• Create positions that are intended to accommodate disabled scholars through expanded recruitment, including positions that are partially and fully remote
	• Actively promote disabled scholars and highlight their work to enhance visibility and representation
	• Enhance mentorship of disabled faculty, students, and staff
	• Use images that reflect range of disability (including visible and invisible) in all media, not just media surrounding disability
Monitoring & evaluation	• Create and maintain accessibility committees to ensure access needs for all are met
	• Maintain high-quality video conferencing integration
	• Maintain and upgrade existing resources
	• Regularly review policies, procedures, and infrastructure using evidence-based evaluation metrics
	• Repeatedly revisit all cells of this table to incorporate new findings and perspectives

^a^The list is not exhaustive, but emphasizes that efforts should be systematic and thorough, from assessment to evaluation.

Our FAM approach extends beyond disability; these general principles also support new parenthood and socio-economic diversity among STEMM practitioners. It also minimizes the need for people with disabilities to claim individual accommodations, which may be met with reluctance, particularly if they are marginalized in other ways, or are unaware of laws and policies regarding accommodations. Our approach is both ethical and efficient, recognizing the vast diversity in the STEMM workforce and embracing that diversity through an inclusive, but not one-size-fits-all, FAM strategy.

## Conclusion

Participation of disabled people is a hallmark of humanity^18^. FAM provides a set of basic principles to enhance participation of people with disabilities in STEMM. Research and funding must be allocated to support the equitable implementation of these principles across the full range of STEMM workplaces. The COVID-19 pandemic has highlighted that many accommodations that once might have seemed extreme are clearly possible. Long COVID reminds us all that no one is more than one illness episode away from lasting disability. The inclusion efforts we have outlined will lead to a post-pandemic life that is more satisfying for everyone. Indeed, the vast majority of readers of this commentary will have been touched by disability, though many are likely unaware of these basic principles of disabled inclusion. We will all benefit both directly and indirectly through closing knowledge gaps and implementing FAM.

## Supplementary information

Supplementary Information
