# Supplementary material for: Community voices: broadening participation in Science, Technology, Engineering, Mathematics, and Medicine among persons with disabilities
Source: Nat Commun. 2022 Dec 3;13:7208. doi: 10.1038/s41467-022-34711-w (PMC9719555; doi:10.1038/s41467-022-34711-w)
Supplement: Supplementary file 1 — Supplementary Information [file 41467_2022_34711_MOESM1_ESM.pdf]

Supplementary Information - Author bios:

Siobhán M. Mattison is an associate professor of evolutionary anthropology at the University of New Mexico. She carries a diagnosis of myasthenia gravis, a rare neuromuscular disorder with pervasive effects on skeletal muscles, as well as other auto-immune conditions. Her research centers on the effects of gender norms on health and well-being. Her passions include science, translational research, and bolstering anti-exclusion efforts in STEMM. This material is based upon work supported by (while serving at) the National Science Foundation. Any opinions, findings, and conclusions or recommendations expressed in this material are those of the author(s) and do not necessarily reflect the views of the National Science Foundation.

Logan Gin is an assistant director of STEM Education in the Sheridan Center for Teaching and Learning at Brown University. He received his PhD in biology education in the School of Life Sciences at Arizona State University where his research focused on the experiences of students with disabilities in evolving science learning environments, such as active learning courses, online instruction, and undergraduate research experiences. He has diastrophic dysplasia dwarfism and uses forearm crutches and a scooter to assist with mobility.

Allistair Abraham is an associate professor of pediatric medicine at George Washington University. His research focuses on treatments for sickle-cell disease. He has amyotrophic lateral sclerosis (ALS), which results in paralysis of skeletal muscles, but does not affect cognition or the ability to treat patients or engage in research.

Megan Moodie is an associate professor of cultural anthropology and current chair of the Faculty Community Networking Group for Faculty with Disabilities and Chronic Illness at the University of California, Santa Cruz. She has Ehlers-Danlos Syndrome Type III and EDS-related dystonia that affects her speech and mobility. Her current project brings together social sciences and the arts to explore gendered experiences of disability and parenting in the United States.

Dr. Oluwaferanmi Okanlami is the Director of Student Accessibility and Accommodation Services at the University of Michigan, where he oversees the office of Services for Students with Disabilities, two Testing Accommodation Centers, and the Adaptive Sports & Fitness Program. He is also an Assistant Professor of Family Medicine, Physical Medicine & Rehabilitation, and Urology at Michigan Medicine, and an Adjunct Assistant Professor of Orthopaedic Surgery at David Geffen School of Medicine at UCLA. "Dr. O" experienced a spinal cord injury during his residency, resulting in incomplete quadriplegia and he now navigates the world as a proud wheelchair user. He speaks on topics related to diversity, equity, and inclusion, including, but not limited to, creating a health system that is accessible to and inclusive of both patients and providers with disabilities, providing reasonable and appropriate accommodations for students with disabilities in higher education. He is passionate about adaptive sports and fitness, striving to provide access to inclusive physical fitness and recreational and competitive sports for all.

Katherine Wander is an associate professor of anthropology at Binghamton University (SUNY). She has epilepsy, which is currently well controlled. Her primary research interest is

evolutionary medicine and public health, particularly around adaptability in human growth and development.
